# Research on the effect of multiple credit ratings from the perspective of financial regulatory systems in Chinese bond market

**DOI:** 10.1371/journal.pone.0312533

**Published:** 2024-11-11

**Authors:** Xiangyun Zhou, Huiling Wang, Luping Zhang

**Affiliations:** 1 Shenzhen Institute of Information Technology, Shenzhen, China; 2 University of Electronic Science and Technology of China, Chengdu, China; University of Barcelona: Universitat de Barcelona, SPAIN

## Abstract

This paper, starting from the effects of financial regulatory policies, considers the interaction between Chengxin_Moody and Lianhe_Fitch with the dual rating system and the multi-rating system, constructs a new ordered Logit model, and attempts to explore the impact of the Notice, the dual rating system and the multiple rating system on the probability of Chinese corporate bond defaults, rating upgrades, rating downgrades, and the magnitude of credit rating migrations. This study compares the effectiveness of different rating regulatory systems. Using nine thousand two hundred and sixty-two data of Chinese corporate bonds as the research samples. Empirical analysis and robustness test reveal the following findings: (1) The issuance of the Notice has a significant positive effect on the implementation of both the dual rating system and the multiple rating system, with a greater impact on the implementation of the multiple rating system; (2) The issuance of the Notice, along with the dual rating system and the multiple rating system, can all reduce the probability of corporate bond defaults, with the multiple rating system showing the best preventive effect against corporate bond defaults; (3) The dual rating system is more effective in promoting rating agencies to adjust rating behaviors, accurately correcting corporate bond ratings and effectively alleviating the issue of rating inflation. Competition among rating agencies intensifies rating shopping; (4) Under the dual rating system, rating agencies are more likely to expand the magnitude of credit rating upgrades and downgrades, enhancing the differentiation between high-quality corporate bonds and junk corporate bonds; (5) The selection of rating information from Chengxin_Moody and Lianhe_Fitch can appropriately adjust the degree of flexibility or tightening in response to rating regulatory systems. Chengxin_Moody demonstrates a more sensitive reaction compared to Lianhe_Fitch regarding the rating regulatory systems. This study provides valuable references for the formulation and evaluation of the effectiveness of Chinese corporate bond rating regulatory policies.

## 1.Introduction

As an essential component of Chinese financial market construction and openness, the bond market serves as a crucial platform for private enterprises and small and medium-sized enterprises to conduct financing and issuance. Credit ratings, as one of the financial infrastructure elements, hold significant importance in ensuring the continuous healthy development of the bond market. However, there have been frequent occurrences of defaults in highly-rated corporate bonds in China, and the phenomena of rating inflation are apparent. In November 2020, state-owned enterprises such as Huachen Automotive Group and Yongmei Group faced unexpected defaults on AAA-rated bonds, revealing the problem of an inverse relationship between bond ratings and default rates. High-rated bond defaults have raised doubts in the market about the accuracy of rating results, leading to increased market volatility and posing systemic risk hazards. Inaccurate ratings exacerbate market information asymmetry, preventing the credit risk warning function that investors rely on from being effectively fulfilled, and may even trigger debt crises and systemic financial risks [1].

To promote the standardized development of the credit rating industry in the bond market, enhance the quality and competitiveness of credit ratings in China, and facilitate the better service of the credit rating industry to the healthy development of the bond market, the People’s Bank of China and other six departments jointly issued the “Guiding Opinions on Promoting the Reform, Opening-up, and High-Quality Development of Corporate Credit Bond Markets” (hereinafter referred to as the “Opinions”) on August 17, 2021. Article 14 of the Opinions encourages issuers to choose active or investor-paid credit ratings, leveraging the cross-validation effects of dual ratings, multiple ratings, and different rating models. On August 6, 2021, the People’s Bank of China, the National Development and Reform Commission, and five other departments jointly issued the “Notice on Promoting the Healthy Development of the Credit Rating Industry in the Bond Market” (hereinafter referred to as the “Notice”). The Notice came into effect on August 6, 2022. Article 10 of the Notice proposes to promote fair competition in the credit rating industry, encourage issuers to choose two or more credit rating agencies to conduct rating business and leverage the cross-validation effects of dual ratings, multiple ratings, and different rating modes.

The release of the Notice has influenced the number of credit ratings for corporate bonds, shifting from single ratings to dual ratings and multiple ratings. As shown in S1 Table, the number of corporate bonds with multiple ratings rapidly increased in 2021, surpassing the number of corporate bonds with dual ratings for the first time. In June 2022, the number of corporate bonds with multiple ratings reached 8666, while the number of corporate bonds with dual ratings was 9568. In July of the same year, the number of corporate bonds with multiple ratings decreased to 7460, and the number of corporate bonds with dual ratings was 8880. It is evident that the Notice which encourages issuers to choose two or more credit rating agencies, has an incentive effect on the rating shopping behavior of issuers and the supervision of corporate bond ratings. This article will further investigates the impact of the implementation of the Notice on the effectiveness of dual ratings and multiple ratings.

According to the Wind database, before the issuance of the Notice, 920 corporate bonds experienced defaults, among which 585 corporate bonds had a bond rating of AA or above, with a high default rate of 63.59%. When the Notice was released, 208 corporate bonds experienced default events, including 87 corporate bonds with a bond rating of AAA, AA+, or AA. Compared with before the issuance of the Notice, the default rate of high-rated corporate bonds has significantly decreased, indicating that the rating regulatory policies can reduce default events of high-rated bonds. Among 890 single-rated corporate bonds, 98 bonds defaulted after the issuance of the Notice. Among 93 dual-rated corporate bonds, 31 bonds defaulted after the issuance of the Notice. Among 143 multiple-rated corporate bonds, 57 bonds defaulted after the issuance of the Notice. This article will discuss whether dual ratings and multiple ratings can prevent corporate bond defaults to some extent.

Jones et al. investigated the regulatory reform in the European credit rating industry and found that regulatory strengthening led to a shift in rating behavior towards conservatism, rather than an improvement in rating quality [2]. The information content of rating downgrades decreased, and the information content of rating upgrades increased. According to the Wind database, from 2014 to 2024, a total of 1531 corporate bonds experienced rating upgrades, and 409 corporate bonds experienced rating downgrades. From the issuance date of the Notice to January 2024, only 43 corporate bonds experienced rating upgrades, including 11 dual-rated bonds and 14 multiple-rated bonds. On the other hand, 67 corporate bonds experienced rating downgrades, including 37 dual-rated bonds and 2 multiple-rated bonds. The issuance of the Notice has significantly reduced the information of rating upgrades for corporate bonds in China, and dual ratings and multiple ratings have different effects on rating adjustments. This article will analyze and compare whether there is a significant difference between dual ratings and multiple ratings in the degree of adjustment for rating upgrades and downgrades of corporate bonds.

Combining the above analysis, the reform of the rating industry regulatory systems has a considerable impact on the implementation of multiple ratings and the rating behaviors of corporate bonds. This article, starting from the perspective of the regulatory systems of corporate bond ratings in China, will explore the following questions: (1) Does the issuance of the Notice significantly affect the implementation of dual ratings and multiple ratings? (2) Can the issuance of the Notice, along with the dual rating system and the multiple rating system, effectively reduce the probability of corporate bond defaults? (3) Do the dual rating system and the multiple rating system have significant impacts on rating upgrades, rating downgrades, and the magnitude of credit rating changes for corporate bonds?

The main structure of this article is as follows: Section 2 reviews relevant important literature. Section 3 introduces moderating variables and constructs a multivariate ordered Logit model. Section 4 empirically analyzes the three questions of this article using the multivariate ordered Logit model, and Section 5 discusses the implications of research results.

## 2. Literature review

Financial markets such as government bond market, stock market, sovereign debt, and corporate bonds emphasize the importance of credit risk assessment [3–5]. Most credit rating studies were based on the premise of rating shopping [6,7]. Characteristics of the rating shopping mechanism are: Issuers attempt to purchase ratings from candidate rating agencies that are more favorable for their own corporate bond issuance to reduce financing costs [8,9]. Rating shopping was an important assumption combined with the rating market structure, and it may led to conflicts of interests among rating agencies [10].

There is a close relationship between rating regulatory mechanisms and rating quality. Utilizing regulatory mechanisms can alleviate the problems of information asymmetry in financial markets. After the enactment of the Dodd-Frank Act, regulations in the use of multiple credit rating reduced the degree of rating inflation, which was related to the increased reliance of regulatory authorities on previously recorded ratings [11,12]. However, after regulatory authorities enacted relevant laws, increasing regulatory pressure on rating agencies had negative spillover effects on companies focusing on credit ratings [13]. Increased regulatory pressure on rating agencies weakened the trend of rating upgrades to gain market share [14]. Few studies have focused on the impact of regulatory systems in Chinese rating industry on the behaviors of rating agencies and rating inflation of corporate bonds.

Scholars hold different opinions on the regulatory effects of dual rating systems and multiple rating systems. The dual rating system is beneficial for improving the differentiation of credit ratings. Gradually expanding the implementation scope of dual rating systems and promoting multiple rating mechanisms were conducive to improving rating rationality and hedging against risks of rating downgrades [15,16]. Multiple ratings had a beneficial effect on reducing IPO under-pricing and filing price corrections [17,18]. Kladakis et al. demonstrated that banks with more ratings could generate more liquidity. Issuing companies could convey more valid rating information to the market through multiple ratings to compensate for the information insufficiency and rating inaccuracies of a single credit rating [19,20]. Hanley et al. found that when multiple ratings were available, bonds were used the second-lowest credit rating for classification. However, some scholars believed that increased competition among rating agencies intensified rating shopping and triggered rating inflation [11,21,22]. Lee and Schantl demonstrated that increased competition among rating agencies led to better rating agencies releasing false rating information [23–25]. Existing research mainly focused on the regulatory effects of dual rating systems and multiple rating systems in other countries, and there was a lack of multidimensional perspective on rating changes, comparing the regulatory effects of dual rating systems and multiple rating systems on Chinese corporate bond ratings.

Scholars have employed various methods such as artificial neural network model, deep learning, quantum forecast methods, deep neural decision trees, decision trees techniques to predict credit rating changes, the adequacy of macroeconomic policy against the risks and the effectiveness of regulation [26–31]. However, some scholars used ordered models to analyse rating changes based on the characteristics of credit rating indicators. Oliveira et al. considered the ordinal nature of credit ratings and used an ordered Logit model to discuss rating determinants [32]. Koerniadi used a fixed-effects regression model to examine the risk-taking behaviors after corporate rating downgrades [33]. Gu et al. studied rating agencies’ adjustments to bond ratings after the Dodd-Frank reform, introducing “soft adjustment” indicators in a semi-parametric ordered model and quantifying it into bond-specific thresholds. This article refers to the above research, employing an ordered Logit model to empirically test the effectiveness of the Notice, dual rating system and multiple rating system [34].

In summary, previous studies provided a foundation for this study in terms of the effectiveness of rating regulatory mechanisms and rating methods, but there is still no exploration from the perspective of regulatory system reform in China’s rating industry on the regulatory effects of dual rating and multiple rating systems on the rating inflation of corporate bonds and the behavior of rating agencies. this article explores the impact of the Notice, the dual rating system and the multiple rating system on rating agencies’ behaviors and the alleviation of rating inflation from the perspectives of corporate bond defaults, rating upgrades, rating downgrades, the difference of rating upgrades, and the difference of rating downgrades in China. The following research hypotheses are proposed: (1) The issuance of the Notice has a significantly positive effect on the dual rating system and the multiple rating system. (2) The issuance of the Notice, the dual rating system, and the multiple rating system can reduce corporate bond defaults. (3) The dual rating system and the multiple rating system have significant effects on rating upgrades, downgrades, and the span of credit rating migrations.

## 3. Model construction

Credit ratings are ordered qualitative indicators, and the traditional linear transformation theory of ratings has flaws. Due to the boundaries in rating symbols, estimation bias can occur even in large samples. Discrete choice models are not required the linear transformation of rating symbols. Instead, they directly transform rating symbols to various truncated intervals, effectively avoiding the issues associated with the linear transformation of ratings. Assuming a continuous unobservable latent variable $y_{it}^{*}$ and observable rating symbols *y*_*it*_, the ordered Logit model can be represented as:

$$y_{it}^{*}=x_{it}^{'}\beta +w_{i}^{'}\lambda +\alpha_{i}+\varepsilon_{it}\;i=1,\ldots ,N\;t=1,\ldots ,T$$ (1)

In Eq (1), $x_{it}^{'}$ is explanatory variable that varies with individuals and time. $w_{i}^{'}$ is dummy variable. *α*_*i*_ represents heterogeneity of financial conditions among issuers. The distribution of error term *ε*_*it*_ is independent of individuals and time. The ordered Logit model assumes the relationship between observable variable *y*_*it*_ and continuous latent variable $y_{it}^{*}$ as follows:

$$y_{it}=\{\begin{array}{ccc} AAA & if & y_{it}^{*}\in \tau_{1}\equiv (-\infty ,c_{1}]\\ AA^{+} & if & y_{it}^{*}\in \tau_{2}\equiv (c_{1},c_{2}]\\ AA & if & y_{it}^{*}\in \tau_{3}\equiv (c_{2},c_{3}]\\ \cdots & \cdots & \cdots \\ CC & if & y_{it}^{*}\in \tau_{20}\equiv (c_{19},c_{20}]\\ C & if & y_{it}^{*}\in \tau_{21}\equiv (c_{20},c_{21}]\\ D & if & y_{it}^{*}\in \tau_{22}\equiv (c_{21},+\infty ) \end{array}$$ (2)

In Eq (2), the number of cut-points *c*_*1*_,*c*_*2*_,*…*,*c*_*21*_ are determined by the number of categories of the observable variable *y*_*it*_. These cut-points are estimated by the maximum likelihood function. According to the properties of ordered Logit model, the distribution of observable variable *y*_*it*_ is determined by the distribution of error term *ε*_*it*_.

If the error term *ε*_*it*_ follows the function of logistic cumulative distribution, it is expressed as:

$$F(\varepsilon_{it}|x_{it}^{'},w_{i}^{'})=F(\varepsilon_{it})=\frac{e^{\varepsilon }}{1+e^{\varepsilon }}=\Lambda (\varepsilon_{it})$$ (3)

In Eq (3), *F*(•) represents the function of logistic cumulative distribution.

## 4. Data

### 4.1 Data selection and descriptive statistics

In this study, data are collected from the Wind database covering the period from January 2014 to January 2024, totaling 10024 Chinese corporate bonds. Bonds with missing data are excluded, resulting in a sample of 9262 corporate bonds for analysis in S1 Data. The research period is set to the issuance of the Notice on August 6, 2021, comparing the effects of the dual rating systems and the multiple rating systems and examining the impact on corporate bond defaults before and after the issuance of the Notice. As shown in S2 and S3 Tables, we demonstrate descriptive statistical analysis of the data.Among issued after the publication of the Notice, corporate bonds are totaling 5375. 1720 bonds have dual ratings, and 1883 bonds have multiple ratings. Only 6 bonds default after the Notice issued.

Corporate bond ratings range from AAA, AA+ to C, D, comprising a total of 22 grades. The ratings were assigned values from 1 for AAA to 22 for D in ascending order [11]. The implementation of rating regulatory policies affects rating upgrades and rating downgrades, as well as the migrations of ratings for corporate bonds. According to the sample data, 1385 corporate bonds experience rating upgrades, while 127 corporate bonds are rating downgrades. The magnitude of rating upgrades show a relatively small difference, whereas the downgrades exhibited a larger difference in migration.

Chinese corporate bond ratings are provided by nine rating agencies, including Chengxin_Moody and Lianhe_Fitch, as well as others like Dagong and Brilliance. Since 2006, Moody’s has held a 49% stake in Chengxin_Moody. Fitch has held a 49% stake in Lianhe_Fitch since 2007. Other domestic rating agencies do not collaborate with international rating agencies. Due to differences in rating modes, accuracy, and reputation between domestic and international rating agencies, Chengxin_Moody and Lianhe_Fitch can be selected as the second or the third rating agencies, leveraging the cross-validation role of dual ratings, multiple ratings, and different rating modes. According to the sample data, 1738 corporate bonds have dual ratings, with 516 bonds using Chengxin_Moody and 363 bonds using Lianhe_Fitch as the second rating. Additionally, 1893 corporate bonds have multiple ratings, with 554 bonds using Chengxin_Moody and 368 bonds using Lianhe_Fitch as the third rating.

### 4.2 Variable definitions

Based on the three questions addressed in this study, dependent and independent variables are defined. The indicators and explanations for both dependent and independent variables are presented in S4 Table.

Profitability, solvency, operational efficiency, and development capability of issuers are important factors influencing corporate bond ratings and regulatory changes [35]. In this study, we select indicators such as return on equity, debt-to-equity ratio, current ratio, inventory turnover rate and main business revenue growth rate for issuers from 2014 to 2024 as control variables to describe the financial heterogeneity of these firms.

## 5. Empirical analysis

### 5.1 Comparison of effects on the dual rating system and the multiple rating system after the publication of the notice

To validate the effects of dual rating system and multiple rating system after the publication of the Notice, following the research approach of [11,24], this paper constructs an ordered Logit model.


$$y_{1}^{*}=\beta_{1}\cdot Notice+\lambda_{1}\cdot Chengxin+\lambda_{2}\cdot Lianhe\_Fitch+\sum_{j=1}^{5}\alpha_{j}\cdot Control_{j}+\varepsilon_{1t}$$ (4)

$$y_{2}^{*}=\beta_{2}\cdot Notice+\lambda_{1}\cdot Chengxin+\lambda_{2}\cdot Lianhe\_Fitch+\sum_{j=1}^{5}\alpha_{j}\cdot Control_{j}+\varepsilon_{2t}$$ (5)

Eqs (4) and (5) can explain the impact of the Notice on the dual rating system and the multiple rating system. Here, $y_{1}^{*}$ and $y_{2}^{*}$ are latent variables representing whether there is a dual rating or a multiple rating, respectively. *β*_1_ and *β*_2_ are the estimated coefficients for the independent variables indicating the issuance of the Notice. *λ*_1_ and *λ*_2_ are the estimated coefficients for the dummy variables indicating whether rating agency is Chengxin_Moody or Lianhe_Fitch. *α*_*j*_ represents the estimated coefficients for the five financial indicators as control variables. The error terms *ε*_1*t*_ and *ε*_2*t*_ follow the function of logistic cumulative distribution.

As shown in S5 Table, the issuance of the Notice has a significantly positive impact on the implementation of both the dual rating system and the multiple rating system, with a greater effect on the implementation of the multiple rating system. The ratings provided by Chengxin_Moody and Lianhe_Fitch have a significantly positive effect on the implementation of both the dual rating system and the multiple rating system. The financial conditions of the issuers exhibit significant differences in the implementation of the rating regulatory systems. The net return on equity, current ratio, and main business revenue growth rate have negative impact on the dual rating system. The current ratio, inventory turnover rate, and main business revenue growth rate have negative impact on the multiple rating system.

### 5.2 Comparison of the impact of the notice, dual rating system, multiple rating system on corporate bond defaults

To compare the impact of the notice, the dual rating system and the multiple rating system on corporate bond defaults, an ordered Logit model is constructed in this study.


$$y_{3}^{*}=\beta_{3}\cdot Notice+\lambda_{1}\cdot Chengxin+\lambda_{2}\cdot Lianhe\_Fitch+\sum_{j=1}^{5}\alpha_{j}\cdot Control_{j}+\varepsilon_{3t}$$ (6)

$$y_{3}^{*}=\beta_{4}\cdot Double\_rating+\lambda_{1}\cdot Chengxin+\lambda_{2}\cdot Lianhe\_Fitch+\sum_{j=1}^{5}\alpha_{j}\cdot Control_{j}+\varepsilon_{4t}$$ (7)

$$y_{3}^{*}=\beta_{5}\cdot Multiple\_rating+\lambda_{1}\cdot Chengxin+\lambda_{2}\cdot Lianhe\_Fitch+\sum_{j=1}^{5}\alpha_{j}\cdot Control_{j}+\varepsilon_{5t}$$ (8)

Eqs (6)–(8) can analyze the impact of the Notice, the dual rating system and the multiple rating system on reducing the probability of corporate bond defaults. Here, $y_{3}^{*}$ is the latent variable representing whether defaults occur on corporate bonds. *β*_3_, *β*_4_ and *β*_5_ are the estimated coefficients for the independent variables indicating whether the Notice is issued, whether there are dual ratings, and whether there are multiple ratings, respectively. *λ*_1_ and *λ*_2_ are the estimated coefficients for the dummy variables indicating whether rating agency is Chengxin_Moody or Lianhe_Fitch. *α*_*j*_ represents the estimated coefficients for the control variables, which include five financial indicators. The error terms *ε*_3*t*_, *ε*_4*t*_ and *ε*_5*t*_ follow the functions of logistic cumulative distribution.

As shown in S6 Table, the estimated coefficients for whether the Notice is issued, whether there are dual ratings, and whether there are multiple ratings are all negative. Through calculations, the issuance of the notice, the dual rating system, and the multiple rating system result in corporate bond default probabilities of 0.0335 ($\frac{e^{-3.3606}}{1+e^{-3.3606}}$), 0.1559 ($\frac{e^{-1.6893}}{1+e^{-1.6893}}$), and 0 ($\frac{e^{-17.7757}}{1+e^{-17.7757}}$), respectively. This indicates that the issuance of rating regulatory systems can prevent corporate bond defaults. The Notice has a significantly negative impact on corporate bond defaults, and the multiple rating system is the most effective in preventing corporate bond defaults. The estimated coefficients for whether bonds rated by Chengxin_Moody and Lianhe_Fitch are both negative, suggesting that the selection of rating information from Chengxin_Moody and Lianhe_Fitch can reduce the probability of corporate bond defaults to zero, but the impacts are not significant.

### 5.3 Comparison of the impact of dual ratings on corporate bond rating upgrades, downgrades, and credit grade migrations

Due to different rating models of Chengxin_Moody and Lianhe_Fitch compared to other domestic rating agencies, selecting information from Chengxin_Moody and Lianhe_Fitch as the second or third rating provides a cross-validation effect. Based on the research of [11], to examine the influence of the dual rating system on rating upgrades, downgrades, and credit grade migrations, and considering the moderating effects of different rating agencies on the rating changes of corporate bonds, two adjusting variables are introduced: Chengxin_Moody * Dual ratings and Lianhe_Fitch * Dual ratings. A new ordered Logit model is constructed to analyze rating information from Chengxin_Moody and Lianhe_Fitch strengthens or weakens the impact of the dual rating system on corporate bond rating changes.


$$y_{4}^{*}=\beta_{6}\cdot Double\_rating+\lambda_{1}\cdot Chengxin+\lambda_{2}\cdot Lianhe\_Fitch+\sum_{j=1}^{5}\alpha_{j}\cdot Control_{j}+\varepsilon_{6t}$$ (9)

$$y_{4}^{*}=\beta_{7}\cdot Double\_rating+\lambda_{1}\cdot Chengxin+\lambda_{2}\cdot Lianhe\_Fitch+\sum_{j=1}^{5}\alpha_{j}\cdot Control_{j}+\varepsilon_{7t}$$ (10)

$$y_{4}^{*}=\beta_{8}\cdot Double\_rating+\lambda_{1}\cdot Lianhe\_Fitch+\lambda_{4}\cdot Lianhe\_Fitch*Double\_rating+\sum_{j=1}^{5}\alpha_{j}\cdot Control_{j}+\varepsilon_{8t}$$ (11)

Eq (9) describes the direct impact of the dual rating system on corporate bond rating upgrades or downgrades, as well as the differences in rating upgrades or downgrades. Eqs (10) and (11) further examine the moderating effect of Chengxin_Moody and Lianhe_Fitch on corporate bond rating changes. Here, $y_{4}^{*}$ is the latent variable representing whether the corporate bond rating is upgraded or downgraded, and the differences in rating upgrades or downgrades. *β*_6_, *β*_7_ and *β*_8_ are the estimated coefficients for the independent variables indicating whether there are dual ratings. *λ*_1_, *λ*_2_, *λ*_3_, *λ*_4_ are the estimated coefficients of dummy variables indicating whether rating agency is Chengxin_Moody or Lianhe_Fitch, Chengxin_Moody * Dual ratings and Lianhe_Fitch * Dual ratings, respectively. *α*_*j*_ represent the estimated coefficients of five financial indicator control variables.The error terms *ε*_6*t*_, *ε*_7*t*_ and *ε*_8*t*_ follow the functions of logistic cumulative distribution.

## 5.3.1 The impact of dual rating system on corporate bond rating upgrades

As shown in S7 Table, the dual rating system has a significant negative impact on corporate bond rating upgrades, indicating that the dual rating system can correct inflated ratings for Chinese corporate bonds, alleviating the issue of rating inflation. Ratings provided by Chengxin_Moody as the second rating has a significantly negative impact on rating upgrades, while ratings provided by Lianhe_Fitch has a significantly positive effect on rating upgrades. This suggests that the choices of different rating agencies by issuers have varying effects on the rating upgrades of corporate bonds. Chengxin_Moody is more cautious in releasing rating upgrades. The debt-to-equity ratio has a significantly negative impact on rating upgrades, while other control variables do not have significant impacts on rating upgrades.

Based on the estimated coefficients of the adjusting variables in S7 Table, the coefficient for Chengxin_Moody * dual ratings is -0.2730, and the coefficient for Lianhe_Fitch * dual ratings is -0.0095. This indicates that rating information provided by Chengxin_Moody and Lianhe_Fitch strengthens the negative relationship between the dual rating system and corporate bond rating upgrades.

## 5.3.2 The impact of dual rating system on corporate bond rating downgrades

As shown in S8 Table, the dual rating system has a significant positive effect on rating downgrades of corporate bonds, indicating that the dual rating system makes it easier for rating agencies to downgrade corporate bond ratings. Rating information provided by Chengxin_Moody as the second rating has a significantly negative impact on rating downgrades, while the impact of Lianhe_Fitch’s information on rating downgrades is not significant, indicating that Chengxin_Moody is more cautious in releasing rating downgrades than Lianhe_Fitch. The return on equity and the current ratio have significantly negative impacts on rating downgrades, while the debt-to-equity ratio has a significantly positive effect on rating downgrades. The inventory turnover rate has no significant impact on rating downgrades. After adding the adjusting variables, the main business revenue growth rate has a significantly negative impact on rating downgrades.

According to the estimated coefficients of the adjusting variables in S8 Table, the coefficient for Chengxin_Moody * Dual ratings is 1.3505, and the coefficient for Lianhe_Fitch * Dual ratings is 0.3006. This indicates that rating information provided by Chengxin_Moody has a significantly promoting effect on positive relationship between the dual rating system and corporate bond rating downgrades, while the impact of Lianhe_Fitch’s rating information on positive relationship between the dual rating system and corporate bond rating downgrades is not significant.

## 5.3.3 The impact of dual rating system on the difference of rating upgrades

As shown in S9 Table, the dual rating system has a significantly positive effect on the difference of rating upgrades. This indicates that the dual rating system encourages rating agencies to more easily increase the range of credit rating upgrades. Rating information provided by Chengxin_Moody has a significantly positive effect on the difference of rating upgrades, while rating information provided by Lianhe_Fitch has a negative impact on the difference of rating upgrades, suggesting that Chengxin_Moody is more likely to increase the amplitude of credit rating upgrades. The debt-to-equity ratio has a significantly positive effect on the difference of rating upgrades, while other control variables have no significant impact on the difference of rating upgrades.

According to the estimated coefficients of adjusting variables in S9 Table, the coefficient of Chengxin_Moody * Dual ratings is 0.2860, and the coefficient of Lianhe_Fitch * Dual ratings is -0.0157. This indicates that rating information provided by Chengxin_Moody strengthens positive relationship between the dual rating system and the difference of rating upgrades. On the other hand, rating information provided by Lianhe_Fitch weakens positive relationship between the dual rating system and the difference of rating upgrades.

## 5.3.4 The impact of dual rating system on the difference of rating downgrades

As shown in S10 Table, the dual rating system has a significant positive effect on the difference of rating downgrades. This indicates that the dual rating system encourages rating agencies to increase the span of credit rating downgrades more easily. Rating information provided by Chengxin_Moody has a significantly negative impact on the difference of rating downgrades, while the impact of rating information provided by Lianhe_Fitch is not significant. This suggests that Chengxin_Moody is more cautious in increasing the magnitude of credit rating downgrades. The return on equity, the main business revenue growth rate, and the current ratio have significantly negative impacts on the difference of rating downgrades, while the asset-liability ratio has a significantly positive impact, and inventory turnover has no significant impact on the difference of rating downgrades.

According to the estimated coefficients of the adjusting variables in S10 Table, the coefficient for Chengxin_Moody * dual ratings is 1.5711, and Lianhe_Fitch * dual ratings is 0.2646. This indicates that rating information provided by Chengxin_Moody has a significantly strengthening effect on positive relationship between the dual rating system and the difference of rating downgrades. However, the moderating effect of rating information provided by Lianhe_Fitch on positive relationship between the dual rating system and the difference of rating downgrades is not significant.

### 5.4 Comparison of the impact of multiple credit ratings on ratings upgrades, rating downgrades, and credit grade migrations of corporate bonds

Similarly, introducing two adjusting variables, namely, Chengxin_Moody * multiple ratings and Lianhe_Fitch * multiple ratings. We constructing a new ordered Logit model to analyze whether rating information provided by Chengxin_Moody and Lianhe_Fitch as the third rating to strengthen or weaken the impact of the multiple credit rating system on the rating changes of corporate bonds.


$$y_{5}^{*}=\beta_{9}\cdot Multiple\_rating+\lambda_{1}\cdot Chengxin+\lambda_{2}\cdot Lianhe\_Fitch+\sum_{j=1}^{5}\alpha_{j}\cdot Control_{j}+\varepsilon_{9t}$$ (12)

$$y_{5}^{*}=\beta_{10}\cdot Multiple\_rating+\lambda_{1}\cdot Chengxin+\lambda_{3}\cdot Chengxin*Multiple\_rating+\sum_{j=1}^{5}\alpha_{j}\cdot Control_{j}+\varepsilon_{10t}$$ (13)

$$y_{5}^{*}=\beta_{11}\cdot Multiple\_rating+\lambda_{1}\cdot Lianhe\_Fitch+\lambda_{4}\cdot Lianhe\_Fitch*Multiple\_rating+\sum_{j=1}^{5}\alpha_{j}\cdot Control_{j}+\varepsilon_{11t}$$ (14)

Eq (12) describes the direct impact of the multiple credit rating system on rating upgrades and rating downgrades of corporate bonds, as well as the difference of rating upgrades and downgrades. Eqs (13) and (14) further verify the moderating effects on Chengxin_Moody and Lianhe_Fitch rating agencies’ rating changes. Here, $y_{5}^{*}$ is the latent variable, representing whether corporate bond ratings are upgraded or downgraded, and the difference of rating upgrades and downgrades. *β*_9_, *β*_10_ and *β*_11_ are the estimated coefficients for whether there is a multiple credit rating. *λ*_1_ to *λ*_4_ are the estimated coefficients for the dummy variables, including whether rating agency is Chengxin_Moody or Lianhe_Fitch, Chengxin_Moody *Multiple ratings and Lianhe_Fitch * Multiple ratings, respectively. *α*_*j*_ are the estimated coefficients for the control variables representing five financial indicators. The error terms *ε*_9*t*_, *ε*_10*t*_ and *ε*_11*t*_ follow the functions of Logistic cumulative distribution.

#### 5.4.1 The impact of multiple credit rating system on rating upgrades

As shown in S11 Table, the multiple credit rating system has a significant negative impact on rating upgrades, indicating that the multiple rating system still alleviates the issue of rating inflation. Rating information provided by Chengxin_Moody as the third rating has a significantly negative impact on the rating upgrades, while the positive effect of rating information provided by Lianhe_Fitch on rating upgrades is not significant, suggesting that Chengxin_Moody is more cautious in releasing rating upgrades. The debt-to-equity ratio has a significantly negative impact on the rating upgrades, and the impact of other control variables on the rating upgrades is not significant.

Based on the estimated coefficients of adjusting variables in S11 Table, the coefficient for Chengxin_Moody * Multiple ratings is 0.0978, and the coefficient for Lianhe_Fitch *Multiple ratings is -0.2679. This indicates that rating information provided by Chengxin_Moody weakens the negative relationship between the multiple rating system and rating upgrades of corporate bond ratings, while rating information provided by Lianhe_Fitch strengthens the negative relationship between the multiple rating system and rating upgrades. After incorporating the adjusting variables, the positive impact of rating information provided by Lianhe_Fitch on the rating upgrades becomes significant, suggesting that considering the interaction between Lianhe_Fitch and the multiple rating system, competition among rating agencies makes Lianhe_Fitch more likely to inflate ratings, catering to the rating shopping behavior of bond issuers.

#### 5.4.2 The impact of multiple rating system on rating downgrades

As shown in S12 Table, the multiple rating system has a significantly negative impact on rating downgrades of corporate bond ratings. This indicates that the multiple rating system promotes rating agencies to be more cautious in downgrading corporate bond ratings. Both rating information provided by Chengxin_Moody and Lianhe_Fitch have negative impacts on rating downgrades, but the effects are not significant. The return on equity, the current ratio, and the main business revenue growth rate have significantly negative impacts on rating downgrades, while the debt-to-equity ratio has a significantly positive impact, and the inventory turnover rate has no significant impact on rating downgrades.

According to the estimated coefficients of adjusting variables in S12 Table, the coefficient for Chengxin_Moody * Multiple ratings is 0.5741, and the coefficient for Lianhe_Fitch * Multiple ratings is 2.3115. This indicates that rating information provided by Lianhe_Fitch significantly weakens positive relationship between the multiple rating system and rating downgrades of corporate bond ratings. However, the impact of Chengxin_Moody’s rating information on the relationship between the multiple rating system and rating downgrades of corporate bond ratings is not significant.

#### 5.4.3 The impact of multiple rating system on the difference of rating upgrades

As shown in S13 Table, the multiple rating system has a significant positive effect on the difference of corporate bond rating upgrades, indicating that the multiple rating system encourages rating agencies to more easily enhance the span of credit rating upgrades. Rating information provided by Chengxin_Moody has a significantly positive effect on the difference of rating upgrades, while rating information provided by Lianhe_Fitch has a negative impact on the difference of rating upgrades, suggesting that Chengxin_Moody is more likely to increase the magnitude of credit rating upgrades. The debt-to-equity ratio has a significantly positive effect on the the difference of rating upgrades, and the effects of other control variables on the difference of rating upgrades are not significant.

Based on the estimated coefficients of adjusting variables in S13 Table, the coefficient for Chengxin_Moody * Multiple ratings is -0.0362, and the coefficient for Lianhe_Fitch * Multiple ratings is 0.3310. This indicates that rating information provided by Chengxin_Moody weakens positive relationship between the dual rating system and the difference of rating upgrades, while rating information provided by Lianhe_Fitch strengthens positive relationship between the dual rating mechanism and the difference of rating upgrades. After incorporating the adjusting variables, the negative impact of Lianhe_Fitch’s rating information on the difference of rating upgrades becomes significant, suggesting that considering the interaction between Lianhe_Fitch and the multiple rating system, the rating regulatory systems encourage Lianhe_Fitch to be more cautious in increasing the magnitude of credit rating upgrades.

#### 5.4.4 The impact of multiple rating system on the difference of rating downgrades

As shown in S14 Table, the multiple rating system has a significantly negative impact on the difference of rating downgrades. This indicates that the multiple rating system encourages rating agencies to be more cautious in increasing the span of credit rating downgrades. Both Chengxin_Moody and Lianhe_Fitch releasing rating information exhibit negative effects on the difference of rating downgrades, although these effects are not statistically significant. The return on equity and the main business revenue growth rate have significantly negative impacts on the the difference of rating downgrades, while the debt-to-equity ratio has a significantly positive impact. The effects of the current ratio and the inventory turnover ratio on the difference of rating downgrades are not statistically significant.

Based on the estimated coefficients of adjusting variables in S14 Table, the coefficient for Chengxin_Moody * Multiple ratings is 0.5587, and the coefficient for Lianhe_Fitch * Multiple ratings is 2.2562. This suggests that rating information provided by Lianhe_Fitch significantly strengthens the negative relationship between the multiple rating system and the difference of rating downgrades. However, the interaction effect of rating information provided by Chengxin_Moody has the negative relationship between the multiple rating system and the difference of rating downgrades is not statistically significant.

### 5.5 Empirical comparison of dual rating system and multiple rating system on corporate bond rating changes

According to empirical analysis based on the results of S7 and S10 Tables, the results reveal that the dual rating system has a greater negative impact on rating upgrades, indicating that the dual rating system is more effective in mitigating the issue of inflated ratings. Under the dual rating system, both Chengxin_Moody and Lianhe_Fitch significantly have influence on rating upgrades, while the impact of Lianhe_Fitch on rating upgrades is not significant under the multiple rating system. This suggests that competition among multiple rating agencies weakens the publication of Lianhe_Fitch’s ratings, making the dual rating system more favorable for Chengxin_Moody and Lianhe_Fitch to adjust their rating behaviors. Considering the interaction among Chengxin_Moody, Lianhe_Fitch, the dual rating system, and the multiple rating system, the publication of Chengxin_Moody’s ratings have a restraining effect on the negative relationship between the multiple rating system and rating upgrades, indicating that competition among multiple rating agencies leads Chengxin_Moody to issue ratings that cater to the rating shopping behaviors of bond issuers.

Due to S8 and S12 Tables, the dual rating system has a significantly positive effect on rating downgrades, while the multiple rating system has a significantly negative impact on rating downgrades. This implies that under the dual rating system, rating agencies are more likely to downgrade ratings, but under the multiple rating system, rating agencies are more cautious about rating downgrades. Under different rating regulatory systems, both Chengxin_Moody and Lianhe_Fitch have negative impacts on rating downgrades. Only under the dual rating system, the negative impact of Chengxin_Moody on rating downgrades is significant. Including the moderating variables, Chengxin_Moody’s ratings have inhibiting effects on the positive relationship between the dual rating system and rating downgrades, while Lianhe_Fitch’s ratings weaken the negative relationship between the multiple rating system and rating downgrades. This indicates that purchasing ratings provided by Chengxin_Moody and Lianhe_Fitch can appropriately adjust the looseness or tightness of the rating regulatory systems regarding rating downgrades.

S9 and S13 Tables reveal that the dual rating system has a significantly greater positive effect on the difference of rating upgrades, indicating that under the dual rating system, rating agencies are more likely to expand the magnitude of credit rating upgrades, causing a gap between high-quality and junk corporate bonds to widen. Under different rating regulatory systems, Chengxin_Moody has a significantly positive effect on the difference of rating upgrades, while the impact of Lianhe_Fitch on the difference of rating upgrades is not significant. This suggests that Chengxin_Moody is more sensitive to reactions of the rating regulatory systems. With the inclusion of moderating variables, the positive correlation between Chengxin_Moody and the difference of rating upgrades under the dual rating system is strengthened, and the positive correlation under the multiple rating system is weakened. Meanwhile, the positive correlation between Lianhe_Fitch and the difference of rating upgrades under the dual rating system is weakened, and the positive correlation under the multiple rating system is strengthened. The above research indicates that under the dual rating system, purchasing Chengxin_Moody ratings is more likely to expand the magnitude of rating upgrades, while under the multiple rating system, purchasing ratings of Chengxin_Moody is more conservative in increasing the difference of rating upgrades. Under different rating regulatory systems, the difference of rating upgrades of Lianhe_Fitch is opposite to that of Chengxin_Moody.

As shown in S10 and S14 Tables, the dual rating system has a significantly positive effect on the difference of rating downgrades, while the multiple rating system has a significantly negative impact on the difference of rating downgrades. This indicates that under the dual rating system, rating agencies are more likely to increase the magnitude of credit rating downgrades, while under the multiple rating system, rating agencies are more cautious in expanding the magnitude of credit rating downgrades. Under the dual rating system, Chengxin_Moody has a significantly negative impact on the difference of rating downgrades, while the impact of Lianhe_Fitch on the difference of rating downgrades is not significant. This suggests that Chengxin_Moody is more conservative in changing the magnitude of rating downgrades. Under the multiple rating system, both Chengxin_Moody and Lianhe_Fitch do not significantly impact the the difference of rating downgrades. Including adjusting variables, the positive correlation between Chengxin_Moody and the difference of rating downgrades under the dual rating system is significantly enhanced, while the positive correlation between Lianhe_Fitch and the difference of rating downgrades under the multiple rating system is significantly weakened. This indicates that purchasing ratings of Chengxin_Moody and Lianhe_Fitch can appropriately adjust the impact of the rating regulatory systems on the magnitude of rating downgrades.

### 5.6 Robustness test

To demonstrate the robustness of empirical results, this study uses a controlled ordered Logit model including no control variables and adjusting variables to directly verify the impact of the Notice on the dual rating system and multiple rating system, the impact of the rating regulatory systems on corporate bond defaults, and the effects of the dual-rating system and multiple rating system on rating upgrades, rating downgrades, the magnitude of rating upgrades, and the magnitude of rating downgrades.

Comparing the empirical results in S15 Table and S5 Table to S14 Table, although the estimated coefficients for the variables of the Notice, the dual rating system and the multiple rating system are changed when control variables and adjusting variables are removed, but the signs and significance levels remain unchanged. This indicates the robustness of empirical results from S5 Table to S14 Table.

## 6. Conclusions

This study focuses on the rating inflation of Chinese corporate bonds. Centered around the effects of financial regulatory reforms in the bond market, we propose and address three research questions. Considering the practical situations of the credit rating industry in China and the interaction between Chengxin_Moody and Lianhe_Fitch with the dual-rating system and multi-rating system, we construct a new ordered Logit model. We attempt to explore the impact of the Notice, the dual-rating system, and the multi-rating system on the probability of corporate bond defaults, rating upgrades, rating downgrades and the magnitude of credit rating changes. The following conclusions are drawn:

Firstly, The issuance of the Notice has a significantly positive effect on the implementation of both the dual-rating system and the multi-rating system, with a greater influence on the latter. The Notice, along with the dual-rating and multi-rating systems, can reduce the probability of corporate bond defaults. The Notice has a significant effect on preventing corporate bond defaults, and the multi-rating system performs the best in preventing such defaults.

Secondly, Both the dual-rating system and the multi-rating system have a significantly negative impact on rating upgrades, with the dual-rating system having a greater negative influence. This indicates that the dual-rating system is more effective in correcting corporate bond ratings and mitigating rating inflation. Under the dual-rating system, Chengxin_Moody has a significantly negative impact on rating upgrades, while Lianhe_Fitch has a significantly positive effect. Under the multi-rating system, Chengxin_Moody has a significantly negative impact on rating upgrades, whereas positive effect of Lianhe_Fitch on rating upgrades is not significant. This suggests that competition among multiple rating agencies weakens rating information provided by Lianhe_Fitch, and the dual-rating system is more favorable for Chengxin_Moody and Lianhe_Fitch to adjust their rating behaviors. Chengxin_Moody is more cautious in rating upgrades. Considering the interaction between Chengxin_Moody and Lianhe_Fitch with the dual-rating system and the multi-rating system, competition among multiple rating agencies leads to increased rating shopping behaviors. The ratings provided by Chengxin_Moody have a significantly strengthening effect on rating downgrades under the dual-rating system.

Thirdly, The dual-rating system has a significantly positive effect on rating downgrades, while the multi-rating system has a significantly negative impact. This implies that under the dual-rating system, rating agencies are more likely to decrease ratings, but under the multi-rating system, rating agencies are more cautious in rating downgrades. Under the dual-rating system, Chengxin_Moody has a significantly negative impact on rating downgrades, indicating that the dual-rating system has a regulatory effect on rating information. There are significant differences in the moderating effects of Chengxin_Moody and Lianhe_Fitch on the relationship between different rating regulatory systems and rating downgrades. This suggests that selecting rating information of Chengxin_Moody and Lianhe_Fitch can appropriately adjust the looseness or tightness of the impact of rating regulatory systems on rating downgrades.

Fourthly, Both the dual-rating system and the multi-rating system have a significantly positive effect on the difference of rating upgrade, with the dual-rating system having a greater positive effect. This indicates that under the dual-rating system, rating agencies are more likely to increase the magnitude of credit rating upgrades, enhancing the differentiation between high-quality and junk corporate bonds. Chengxin_Moody is more sensitive to rating changes in regulatory systems than Lianhe_Fitch. Under different rating regulatory systems, Lianhe_Fitch moderates the changes in the behaviors of the difference of rating upgrade compared to Chengxin_Moody.

Fifthly, The dual-rating system has a significantly positive effect on the difference of rating downgrade, while the multi-rating system has a significantly negative impact. This implies that under the dual-rating system, rating agencies are more likely to increase the magnitude of rating downgrades, but under the multi-rating system, rating agencies are more cautious in expanding the magnitude of credit rating downgrades. Chengxin_Moody is more conservative in the difference of rating downgrades. Selecting rating information of Chengxin_Moody and Lianhe_Fitch can appropriately adjust the impact of rating regulatory systems on the magnitude of rating downgrades.

## Supporting information

S1 Data This is the data set for the research. (XLSX)

S1 Table This is the number of corporate bonds with several ratings. (DOC)

S2 Table This is the descriptive statistics before the issuance of the Notice. (DOC)

S3 Table This is the descriptive statistics after the issuance of the Notice. (DOC)

S4 Table This is the selection and description of variables. (DOC)

S5 Table This is comparison of effects on rating regulatory systems after the publication of the Notice. (DOC)

S6 Table This is the impact of rating regulatory systems on corporate bond defaults. (DOC)

S7 Table This is the impact of dual rating system on corporate bond rating upgrades. (DOC)

S8 Table This is the impact of dual rating system on corporate bond rating downgrades. (DOC)

S9 Table This is the impact of dual rating system on the difference of rating upgrades. (DOC)

S10 Table This is the impact of dual rating system on the difference of rating downgrades. (DOC)

S11 Table This is the impact of multiple rating system on corporate bond rating upgrades. (DOC)

S12 Table This is the impact of multiple rating system on corporate bond rating downgrades. (DOC)

S13 Table This is the impact of multiple rating system on the difference of rating upgrades. (DOC)

S14 Table This is the impact of multiple rating system on the difference of rating downgrades. (DOC)

S15 Table This is the empirical results of robustness test. (DOC)
