# Supplementary material for: Research on the effect of multiple credit ratings from the perspective of financial regulatory systems in Chinese bond market
Source: PLoS One. 2024 Nov 11;19(11):e0312533. doi: 10.1371/journal.pone.0312533 (PMC11554074; doi:10.1371/journal.pone.0312533)
Supplement: S1 Table — (DOC) [file pone.0312533.s002.doc]

**Table 1**

Table 1 is the number of corporate bonds with several ratings.

This table presents the number of corporate bonds with dual ratings and multiple ratings from 2017 to 2023.

| Year | The number of corporate bonds with dual ratings | The number of corporate bonds with multiple ratings |
| --- | --- | --- |
| 2023 | 965 | 1 |
| 2022 | 20378 | 16976 |
| 2021 | 855 | 2943 |
| 2020 | 125 | 38 |
| 2019 | 45 | 12 |
| 2018 | 14 | 4 |
| 2017 | 0 | 1 |
| Total | 22382 | 19975 |

Data sources: Wind database
