# Supplementary material for: Research on the effect of multiple credit ratings from the perspective of financial regulatory systems in Chinese bond market
Source: PLoS One. 2024 Nov 11;19(11):e0312533. doi: 10.1371/journal.pone.0312533 (PMC11554074; doi:10.1371/journal.pone.0312533)
Supplement: S2 Table — (DOC) [file pone.0312533.s003.doc]

**Table 2**

Table 2 is the descriptive statistics before the issuance of the Notice

| Variables | Minimum | Maximum | Mean | Std. Deviation |
| --- | --- | --- | --- | --- |
| Rating upgrades | 0 | 1 | 0.2400 | 0.4290 |
| The scores of rating upgrades | 0 | 3 | 0.3900 | 0.7410 |
| the difference of rating upgrades | -3 | 1 | -0.2500 | 0.4640 |
| Rating downgrades | 0 | 1 | 0.0200 | 0.1230 |
| The scores of rating downgrades | 0 | 21 | 0.1200 | 1.3450 |
| The difference of rating downgrades | 0 | 20 | 0.0900 | 1.1460 |
| Dual ratings | 0 | 1 | 0 | 0.0680 |
| Multiple ratings | 0 | 1 | 0 | 0.0510 |
| Chengxin_Moody | 0 | 1 | 0.1700 | 0.3780 |
| Lianhe_Fitch | 0 | 1 | 0.1600 | 0.3640 |
| Return on equity | -709.7962 | 127.7334 | 1.8482 | 21.2721 |
| Debt-to-equity ratio | 0 | 269.6334 | 56.1006 | 15.7473 |
| Current ratio | 0 | 112.5106 | 3.5074 | 4.8958 |
| Inventory turnover rate | 0 | 9976.6024 | 11.0254 | 238.5865 |
| Main business revenue growth rate | -99.9329 | 277866.8624 | 96.6545 | 4.4608 |
| Corporate bond defaults | 0 | 1 | 0 | 0.0530 |
| Valid N | 3887 | | | |

Data sources: Wind database
