# Supplementary material for: Research on the effect of multiple credit ratings from the perspective of financial regulatory systems in Chinese bond market
Source: PLoS One. 2024 Nov 11;19(11):e0312533. doi: 10.1371/journal.pone.0312533 (PMC11554074; doi:10.1371/journal.pone.0312533)
Supplement: S3 Table — (DOC) [file pone.0312533.s004.doc]

**Table 3**

Table 3 is the descriptive statistics after the issuance of the Notice

| Variables | Minimum | Maximum | Mean | Std. Deviation |
| --- | --- | --- | --- | --- |
| Rating upgrades | 0 | 1 | 0.0800 | 0.2750 |
| The scores of rating upgrades | 0 | 3 | 0.1100 | 0.3750 |
| the difference of rating upgrades | -2 | 0 | -0.0800 | 0.2810 |
| Rating downgrades | 0 | 1 | 0.0100 | 0.1110 |
| The scores of rating downgrades | 0 | 21 | 0.0700 | 0.8270 |
| The difference of rating downgrades | 0 | 20 | 0.0400 | 0.6880 |
| Dual ratings | 0 | 1 | 0.3200 | 0.4670 |
| Multiple ratings | 0 | 1 | 0.3500 | 0.4770 |
| Chengxin_Moody | 0 | 1 | 0.2300 | 0.4240 |
| Lianhe_Fitch | 0 | 1 | 0.1600 | 0.3650 |
| Return on equity | -54.7634 | 30.4201 | 1.9978 | 2.9770 |
| Debt-to-equity ratio | 5.2309 | 296.8173 | 55.4560 | 14.4753 |
| Current ratio | 0.1841 | 3148.8084 | 4.2803 | 43.0043 |
| Inventory turnover rate | 0 | 1926.5754 | 4.6058 | 59.7207 |
| Main business revenue growth rate | -92.3682 | 910.2653 | 18.2258 | 43.2601 |
| Corporate bond defaults | 0 | 1 | 0 | 0.0530 |
| Valid N | 5375 | | | |

Data sources: Wind database
