# Supplementary material for: Research on the effect of multiple credit ratings from the perspective of financial regulatory systems in Chinese bond market
Source: PLoS One. 2024 Nov 11;19(11):e0312533. doi: 10.1371/journal.pone.0312533 (PMC11554074; doi:10.1371/journal.pone.0312533)
Supplement: S4 Table — (DOC) [file pone.0312533.s005.doc]

**Table 4**

Table 4 is the selection and description of variables.

This table reports three research questions and the explanations of all variables.

| Question 1：Does the issuance of the Notice have a significant impact on the implementation of the dual rating system and the multiple rating system? | | | |
| --- | --- | --- | --- |
| dependent variables | description | Independent variables | description |
| dual ratings | Dummy variable. If two rating agencies issue rating information for corporate bonds with a value of 1, otherwise it is 0. | The Notice | Dummy variable. If corporate bonds are issued from the date of the Notice with a value of 1, otherwise it is 0. |
| multiple ratings | Dummy variable. If three rating agencies issue rating information for corporate bonds with a value of 1, otherwise it is 0. | Chengxin_Moody | Dummy variable. If one of ratings of corporate bonds is provided by Chengxin_Moody Rating Agency with a value of 1, otherwise it is 0. |
|  |  | Lianhe_Fitch | Dummy variable. If one of ratings of corporate bonds is provided by Lianhe_Fitch Rating Agency with a value of 1, otherwise it is 0. |
| Question 2：Do the issuance of the Notice, the dual rating system and the multiple rating system effectively reduce the probability of corporate bond defaults? | | | |
| dependent variables | description | Independent variables | description |
| Corporate bond defaults | Dummy variable. If a default event occurs on corporate bonds, the value is 1, otherwise it is 0. | The Notice | Dummy variable. If corporate bonds are issued from the date of the Notice with a value of 1, otherwise it is 0. |
|  |  | dual ratings | Dummy variable. If two rating agencies issue rating information for corporate bonds with a value of 1, otherwise it is 0. |
|  |  | multiple ratings | Dummy variable. If three rating agencies issue rating information for corporate bonds with a value of 1, otherwise it is 0. |
| Question 3：Do the dual rating system and multiple rating system have a significant impact on the rating upgrades and rating downgrades of corporate bonds, as well as the magnitude of rating changes? | | | |
| dependent variables | description | Independent variables | description |
| rating upgrades | Dummy variable. If ratings increase with a value of 1, otherwise it is 0. | dual ratings | Dummy variable. If two rating agencies issue rating information for corporate bonds with a value of 1, otherwise it is 0. |
| rating downgrades | Dummy variable. If ratings decrease with a value of 1, otherwise it is 0. | multiple ratings | Dummy variable. If three rating agencies issue rating information for corporate bonds with a value of 1, otherwise it is 0. |
| The difference of rating upgrades | The value of rating upgrades minus the original ratings | Chengxin_Moody | Dummy variable. If one of ratings of corporate bonds is provided by Chengxin_Moody rating agency with a value of 1, otherwise it is 0. |
| The difference of rating downgrades | The value of rating downgrades minus the original ratings | Lianhe_Fitch | Dummy variable. If one of ratings of corporate bonds is provided by Lianhe_Fitch rating agency with a value of 1, otherwise it is 0. |
