# Supplementary material for: Research on the effect of multiple credit ratings from the perspective of financial regulatory systems in Chinese bond market
Source: PLoS One. 2024 Nov 11;19(11):e0312533. doi: 10.1371/journal.pone.0312533 (PMC11554074; doi:10.1371/journal.pone.0312533)
Supplement: S5 Table — (DOC) [file pone.0312533.s006.doc]

**Table 5**

Table 5 is comparison of effects on rating regulatory systems after the publication of

the Notice.

This table reports the empirical results of effects on the dual rating system and the multiple rating system before and after the issuance of the Notice.

| Variables | The dual rating system | The multiple rating system |
| --- | --- | --- |
|  | Ordered Logit (1) | Ordered Logit (2) |
| The Notice | 4.6665***  (0.2416) | 5.5231***  （0.3228） |
| Chengxin_Moody | 0.6689***  (0.0701) | 0.5392***  （0.0696） |
| Lianhe_Fitch | 0.7204***  (0.0804) | 0.5001***  （0.0805） |
| Return on equity | -0.0038  (0.0033) | 0.0266***  （0.0089） |
| Debt-to-equity ratio | 0.0040*  (0.0021) | 0.0205***  （0.0026） |
| Current ratio | -0.0049  (0.0110) | -0.0636***  （0.0136） |
| Inventory turnover rate | 0.0004**  (0.0002) | -0.0015  （0.0009） |
| Main business revenue growth rate | -0.0041***  (0.0009) | -0.0005  （0.0007） |
| *C_1_* | 5.8378  (0.2796) | 7.3175  （0.3809） |

***、**、*denote that the coefficient is statistically significant at the 10%, 5%, 1% levels respectively.
