# Supplementary material for: Research on the effect of multiple credit ratings from the perspective of financial regulatory systems in Chinese bond market
Source: PLoS One. 2024 Nov 11;19(11):e0312533. doi: 10.1371/journal.pone.0312533 (PMC11554074; doi:10.1371/journal.pone.0312533)
Supplement: S6 Table — (DOC) [file pone.0312533.s007.doc]

**Table 6**

Table 6 is the impact of rating regulatory systems on corporate bond defaults.

This table reports the impact of rating regulatory systems on corporate bond defaults before and after the Notice, the dual rating system and the multiple rating system.

| Variables | Corporate bond defaults | | |
| --- | --- | --- | --- |
|  | Ordered Logit (1) | Ordered Logit (2) | Ordered Logit (3) |
| The Notice | -3.3606*  (1.8023) |  |  |
| Dual ratings |  | -1.6893  (1.9747) |  |
| Multiple ratings |  |  | -17.7757  (6078.9030) |
| Chengxin_Moody | -17.6006  (4487.4060) | -16.6351  (2519.5370) | -18.0131  (4928.6190) |
| Lianhe_Fitch | -17.8148  (5462.2010) | -16.6546  (3139.0670) | -18.1962  (6799.9580) |
| Return on equity | 0.0269  (0.0605) | 0.0388  (0.0645) | 0.0416  (0.0631) |
| Debt-to-equity ratio | 0.0534***  (0.0091) | 0.0545***  (0.0112) | 0.0508***  (0.0116) |
| Current ratio | 0.0004  (0.0066) | -0.0005  (0.0071) | -0.0006  (0.0076) |
| Inventory turnover rate | -0.0008  (0.0134) | -0.0008  (0.0149) | -0.0009  (0.0129) |
| Main business revenue growth rate | -0.0021  (0.0076) | -0.0045  (0.0102) | -0.0048  (0.0103) |
| *C_1_* | 9.2869  (0.8281) | 9.9549  (0.9421) | 9.6309  (0.9673) |

***、**、*denote that the coefficient is statistically significant at the 10%, 5%, 1% levels respectively.
