# Supplementary material for: Research on the effect of multiple credit ratings from the perspective of financial regulatory systems in Chinese bond market
Source: PLoS One. 2024 Nov 11;19(11):e0312533. doi: 10.1371/journal.pone.0312533 (PMC11554074; doi:10.1371/journal.pone.0312533)
Supplement: S7 Table — (DOC) [file pone.0312533.s008.doc]

**Table 7**

Table 7 is the impact of dual rating system on corporate bond rating upgrades.

This table reports the effect of the dual rating system on rating upgrades and the rating behaviors of Chengxin_Moody and Lianhe_Fitch.

| Variables | Rating upgrades | | |
| --- | --- | --- | --- |
|  | Ordered Logit (1) | Ordered Logit (2) | Ordered Logit (3) |
| Dual ratings | -1.0994***  (0.1039) | -1.0223***  (0.1169) | -1.1176***  (0.1176) |
| Chengxin_Moody * Dual ratings |  | -0.2730  (0.2510) |  |
| Lianhe_Fitch * Dual ratings |  |  | -0.0095  (0.2476) |
| Chengxin_Moody | -0.1724**  (0.0796) | -0.1703**  (0.0828) |  |
| Lianhe_Fitch | 0.1402*  (0.0814) |  | 0.1816**  (0.0844) |
| Return on equity | 0.0017  (0.0032) | 0.0018  (0.0032) | 0.0015  (0.0031) |
| Debt-to-equity ratio | -0.0060***  (0.0022) | -0.0057**  (0.0023) | -0.0063***  (0.0022) |
| Current ratio | -0.0012  (0.0044) | -0.0015  (0.0057) | -0.0010  (0.0033) |
| Inventory turnover rate | -0.0001  (0.0003) | -0.0001  (0.0003) | -0.0001  (0.0003) |
| Main business revenue growth rate | -0.0003  (0.0003) | -0.0003  (0.0003) | -0.0003  (0.0004) |
| *C_1_* | 1.2420  (0.1307) | 1.2362  (0.1343) | 1.2585  (0.1278) |

***、**、*denote that the coefficient is statistically significant at the 10%, 5%, 1% levels respectively.
