# Supplementary material for: Research on the effect of multiple credit ratings from the perspective of financial regulatory systems in Chinese bond market
Source: PLoS One. 2024 Nov 11;19(11):e0312533. doi: 10.1371/journal.pone.0312533 (PMC11554074; doi:10.1371/journal.pone.0312533)
Supplement: S8 Table — (DOC) [file pone.0312533.s009.doc]

**Table 8**

Table 8 is the impact of dual rating system on corporate bond rating downgrades.

This table reports the effect of the dual rating system on rating downgrades and the rating behaviors of Chengxin_Moody and Lianhe_Fitch.

| Variables | Rating downgrades | | |
| --- | --- | --- | --- |
|  | Ordered Logit (1) | Ordered Logit (2) | Ordered Logit (3) |
| Dual ratings | 0.9588***  (0.2006) | 0.7195***  (0.2266) | 0.8427***  (0.2242) |
| Chengxin_Moody * Dual ratings |  | 1.3505**  (0.5826) |  |
| Lianhe_Fitch * Dual ratings |  |  | 0.3006  (0.4975) |
| Chengxin_Moody | -0.6037**  (0.2741) | -1.2691**  (0.4658) |  |
| Lianhe_Fitch | -0.2021  (0.2532) |  | -0.1777  (0.3300) |
| Return on equity | -0.0050***  (0.0016) | -0.0050***  (0.0016) | -0.0052***  (0.0016) |
| Debt-to-equity ratio | 0.0215***  (0.0046) | 0.0222***  (0.0045) | 0.0222***  (0.0045) |
| Current ratio | -0.1380**  (0.0546) | -0.1355**  (0.0536) | -0.1211***  (0.0528) |
| Inventory turnover rate | -0.0001  (0.0003) | -0.0011  (0.0029) | -0.0011  (0.0029) |
| Main business revenue growth rate | -0.0011  (0.0030) | -0.0172***  (0.0033) | -0.0179***  (0.0034) |
| *C_1_* | 5.1438  (0.3820) | 5.1633  (0.3678) | 5.3341  (0.3669) |

***、**、*denote that the coefficient is statistically significant at the 10%, 5%, 1% levels respectively.

|  |
| --- |
