# Supplementary material for: Research on the effect of multiple credit ratings from the perspective of financial regulatory systems in Chinese bond market
Source: PLoS One. 2024 Nov 11;19(11):e0312533. doi: 10.1371/journal.pone.0312533 (PMC11554074; doi:10.1371/journal.pone.0312533)
Supplement: S9 Table — (DOC) [file pone.0312533.s010.doc]

**Table 9**

Table 9 is the impact of dual rating system on the difference of rating upgrades.

This table reports the effect of the dual rating system on the difference of rating upgrades and the rating behaviors of Chengxin_Moody and Lianhe_Fitch.

| Variables | The difference of rating upgrades | | |
| --- | --- | --- | --- |
|  | Ordered Logit (1) | Ordered Logit (2) | Ordered Logit (3) |
| Dual ratings | 1.0673***  (0.1034) | 0.9877***  (0.1164) | 1.0899***  (0.1169) |
| Chengxin_Moody * Dual ratings |  | 0.2860  (0.2486) |  |
| Lianhe_Fitch * Dual ratings |  |  | -0.0157  (0.2467) |
| Chengxin_Moody | 0.1600**  (0.0797) | 0.1532*  (0.0824) |  |
| Lianhe_Fitch | -0.1297  (0.0821) |  | -0.1653*  (0.0853) |
| Return on equity | -0.0016  (0.0031) | -0.0016  (0.0031) | -0.0014  (0.0030) |
| Debt-to-equity ratio | 0.0061***  (0.0022) | 0.0059***  (0.0022) | 0.0065***  (0.0022) |
| Current ratio | 0.0012  (0.0027) | 0.0013  (0.0026) | 0.0011  (0.0028) |
| Inventory turnover rate | 0.0001  (0.0003) | 0.0001  (0.0002) | 0.0001  (0.0003) |
| Main business revenue growth rate | 0.00002  (0.0030) | 0.00002  (0.00003) | 0.00002  (0.00003) |
| *C_1_* | -7.2661  (0.5150) | -7.2623  (0.5149) | -7.2802  (0.5151) |
| *C_2_* | -4.6956  (0.1857) | -4.6918  (0.1853) | -4.7097  (0.1858) |
| *C_3_* | -1.2613  (0.1270) | -1.2578  (0.1264) | -1.2758  (0.1271) |
| *C_4_* | 9.1305  (0.7248) | 9.1434  (0.7254) | 9.1119  (0.7242) |

***、**、*denote that the coefficient is statistically significant at the 10%, 5%, 1% levels respectively.
