# Supplementary material for: Research on the effect of multiple credit ratings from the perspective of financial regulatory systems in Chinese bond market
Source: PLoS One. 2024 Nov 11;19(11):e0312533. doi: 10.1371/journal.pone.0312533 (PMC11554074; doi:10.1371/journal.pone.0312533)
Supplement: S15 Table — (DOC) [file pone.0312533.s016.doc]

**Table 15**

Table 15 is the empirical results of robustness test.

This table reports the robustness tests of the Notice, the dual rating system and the multiple rating system.

|  | Dual ratings | Multiple ratings | Corporate bond defaults | Rating upgrades | Rating downgrades | The difference of rating upgrades | The difference of rating downgrades |
| --- | --- | --- | --- | --- | --- | --- | --- |
| The Notice | 4.6166***  （0.2381） | 5.3426***  （0.3179） | -0.9320*  （0.5080） | - | - | - | - |
| Dual ratings | - | - | -1.8269  （0.4935） | -1.1052***  （0.1032） | 1.0187***  （0.1843） | 1.0752***  （0.1027） | 1.0760***  （0.1900） |
| Multiple ratings | - | - | -15.7324  （1246.5280） | -0.9171***  （0.0926） | -1.6583***  （0.4190） | 0.9266***  （0.0934） | -1.5814***  （0.4198） |

***、**、*denote that the coefficient is statistically significant at the 10%, 5%, 1% levels respectively.
